# Linking Transformational Leadership with Nurse-Assessed Adverse Patient Outcomes and the Quality of Care: Assessing the Role of Job Satisfaction and Structural Empowerment

**DOI:** 10.3390/ijerph16132381

**Published:** 2019-07-04

**Authors:** Muhammad Asif, Arif Jameel, Abid Hussain, Jinsoo Hwang, Noman Sahito

**Affiliations:** 1 School of Public Affairs, Zijingang Campus, Zhejiang University, Hangzhou 310058, China; 2 The College of Hospitality and Tourism Management, Sejong University, 98 Gunja-Dong, Gwanjin-Gu, Seoul 143-747, Korea; 3 Department of City & Regional Planning, Mehran University of Engineering & Technology, Jamshoro 76062, Pakistan

**Keywords:** transformational leadership, structural empowerment, job satisfaction, nurse-assessed adverse patient outcomes, quality of care, government hospitals, Pakistan

## Abstract

The purpose of this study was to examine the relationships between transformational leadership (TL), structural empowerment (SE), job satisfaction (JS), nurse-assessed adverse patient outcomes (APO), and the quality of care (QOC). The study further investigates the mediating effects of SE and JS on TL-APO and TL-QOC relationships. A total of 600 nurses working at 17 government hospitals in Pakistan completed the survey. The hypothesized model was tested using a confirmatory factor analysis and structural equation modeling. We found a positive relationship between TL, SE, JS, and QOC but negative relationships between TL and APO, SE and APO, and JS and APO. Our study further suggests that SE and JS strongly mediate both TL-APO and TL-QOC relationships.

## 1. Introduction

Patient outcomes and patient care quality are considered as noteworthy elements for health care bodies in the world. A lot of research across the world has exhibited that healthcare schemes are inclined to be faulty, and the danger of adverse patient outcomes (APO) are significant [1,2]. APO is described as the accidental complications caused by healthcare administration rather than patients’ disease procedures, which ultimately result in lengthy hospital stays, increased death ratios, and disability [3]. For instance, more than 98,000 patients die and up to one million more are wounded or injured every year in the USA as a result of avoidable medical errors [2].

Similarly, up to 138,000 hospitalizations occurred during the period from 2014 to 2015 in Canada, and around 30,000 patients agonized through avoidable damage [4]. In Pakistan, around 500,000 individuals, which mainly includes children and women, die every year due to medical errors that include incorrect prescriptions, over medicating, self-treatments, and the adversarial results of medicines [5]. It is also exhibited that a country’s economic cost is also increased due to these APOs, For example, the cost of these APOs in Canada was projected at 1.1 billion US Dollars for the period from 2009 to 2010 [6]. 

Prior research shows that a terrifyingly high ratio of adverse incidents in healthcare institutions is because of preventable circumstances, some of which are due to nurse-related aspects [7]. For example, in a study conducted in five countries, Aiken, et al. [8] concluded that poor working conditions for nurses and inadequate staffing were predictors of adverse patient outcomes. Scholars have associated these outcomes with the low quality of the nursing job atmosphere and the absence of appropriate leadership styles [7,9]. Previous studies found that deficiencies in hospital care quality are common in all countries [10,11] and an improvement in the work environment is a low-cost strategy to improve safety and quality in hospital care and to increase patient satisfaction [12]. In the institutional context, transformational leadership (TL) is known as a relational leadership that helps healthcare organizations to overcome APOs [13]. However, only a few types of research has explored the ways that leadership effects the behavior of the employees and successive implications about the safety consequences of the patients [14,15]. For example, Houser [16] found that transformational leadership practices were positively related to staff expertise and negatively related to staff turnover, both of which contributed to reduced adverse patient outcomes including patient mortality, hospital acquired infections, medication errors and patient falls. Capuano, et al. [17] added to these findings by showing that transformational leadership practices were also associated with staff expertise, which in turn decreased the same adverse patient outcomes. The findings of Paquet, et al. [18] also revealed the indirect effect of leadership on patient outcomes (decreased medication errors and patient length of stay) through reduced absenteeism. On the other hand, Lavoie-Tremblay, et al. [19] indicated that transformational leadership practices potentially lead to high quality of care and weak intention to quit the healthcare facilities. In the perspective of these studies, the main focus was to determine the ways that TL related to factors that affect the quality of healthcare and the safety of patient outcomes. Hence, the aim of this research is to develop a research model that links transformational leadership (TL) to structural empowerment (SE) and job satisfaction (JS) and then to the occurrence of APOs and the patient’s quality of care (QOC). In this research, the researchers assessed how TL impacts the patients’ desired QOC and APO through mediators, SE and JS.

Since TL directly influences workers’ behaviors and achieves the required performance concerning the expectations of their followers [20], previous research has revealed that TL is a crucial leadership style that helps make a helpful work atmosphere where the nursing staff is structurally empowered and highly satisfied to deliver the best patient care [13]. Scholars have recommended that TL should appear as the most relevant leadership style for current stressed and turbulent healthcare work environments [21,22]. Adopting TL to resolve these problems may offer an insight into the healthcare system, therefore leadership may enhance the desired patient outcomes and QOC.

## 2. Theory and Hypotheses

### 2.1. Relationship between Transformational Leadership, Job Satisfaction, and Structural Empowerment

TL is a type of relational leadership where the leaders have respect and trust from their followers and put extra efforts to accomplish institutional objectives [23]. TL has four key components. The first component is idealized influence, which depicts a leader who acts as a role model for his subordinates, establishes extraordinary values of conduct, and express organization vision in order to gain employees’ confidence and trust. The second component, inspirational motivation, reveals a leader’s abilities to express an organizational vision through images, symbols, and signs [23] to motivate their subordinates to perform in a better way. The third component, intellectual stimulation, indicates the level of a leader’s capabilities to inquire the issues faced by their subordinates and generate a wide range of ideas to address these issues and make them available in the decision making process [23]. The final component, individualized consideration, involves leaders seeking the employees’ differences and then facilitate them with a mentor for proper guidance, training, and support in order to reduce the issues and attain their maximum capacity [24]. 

TL has constantly been associated with worker behaviors in healthcare organizations. Scholars propose that the four aspects of transformational managers may act as precursors to make structurally empowering job atmospheres. For example, a transformational leader may encourage nurses using intellectual stimulation by involving them in the process of making decisions, which promotes rational thinking and the growth of knowledge, attitudes, and abilities. These types of leaders’ build stimulated situations for the nursing staff by providing the required assistance, resources, and information at the workplace. Moreover, TL is linked to the employees’ JS [25], institutional performance, employee work commitment [26], and followers’ readiness to put in extra efforts to obtain the assigned goals. McCutcheon, et al. [27] found a significant relationship between TL behaviors and JS among the nurses in Canadian acute care hospitals.

Transformational leaders develop the patient’s care quality and citizenship behaviors by providing an appropriate practicing atmosphere. These studies show the significance of TL to make a work atmosphere that helps and promotes a better practicing environment for nurses to be professional and enhance the desired outcomes for both the nurses and the patients [28]. By creating progressive relationships, transformational managers obtain the confidence of their employees and foresee their requirements by making them structurally empowered in terms of information, professional support, and resources, which ultimately leads to work satisfaction and enhanced QOC. Based on these arguments, we made the following predictions.

**Hypothesis 1** **(H1).** *TL is positively related to nurses’ JS.*

**Hypothesis 2** **(H2).** *TL is positively related to SE.*

### 2.2. Relationship between Structural Empowerment, Job Satisfaction, Quality of Care, and Adverse Patient Outcomes 

The SE theory describes how managers or leaders affect the followers’ behaviors in order to complete their tasks in effective and efficient manners. Transformational leaders can get jobs completed by their followers by granting them access to four basic organizational factors that include resources, opportunities, support, and information. Accessibility to resources denotes having the required stuff, money, stocks, time, and the tools needed to complete the task [29]. Accessibility to opportunities involves incentives, challenges, job position/status, value, and competency appreciation that enhance the abilities and skills of the workers. Support accessibility comprises of the directions and feedback offered by bosses, juniors, managers, and the societal and emotional care from the coworkers. Lastly, accessibility to information involves having adequate information about the institutional objectives, the norms, and the policies along with the technical knowledge required to fulfill the job requirements [29,30]. 

The basic purpose of every healthcare organization especially in terms of nursing care is to fulfill the patient’s needs and achieve the desired patient outcomes [31]. Patient outcome studies have recognized that most of APOs have occurred due to an inappropriate work environment [32] and the absence of effectual and operative leadership [2,9]. According to Aiken, Clarke, Sloane, Sochalski, Busse, Clarke, Giovannetti, Hunt, Rafferty and Shamian [8] and Laschinger and Leiter [33], bad working conditions and a lack of nursing staff are the key factors of APOs, which include rescue failure medication errors and mortality. This research measured different APOs assessed by nurses that include patient falls, medication faults, hospital-assimilated diseases, and pressure sores/bedsores. The nursing-rated quality of healthcare offers the most relevant yet distinctive information about outcomes of the patient, because the nursing staff is concerned virtually at every stage of the patient’s health care, which builds their perception and provides valuable information. McHugh and Stimpfel [34] discovered that nursing-measured QOC was connected with impartial hospital quality indicators, such as the satisfaction of the patient, flop to rescue, and the death rates, offer that the nursing-assessed patients’ outcomes and the real ones are interlinked.

The term JS is described as the optimistic individual perception towards his job and job experiences [35]. It has been linked with worker turnover, service quality and effectiveness, and patient’s satisfaction [36,37]. Patient’s care is the primary focus for nursing staff, and it is a prerequisite for nurses to offer good QOC. Also, it effects their JS in return [38,39,40,41]. These investigations further stipulate evidence and support for the importance of nursing skills provided to patients during their stay in the hospitals with an improved QOC.

Several types of research have recognized the SE concept given by Kanter with diverse nursing staff populations and backgrounds. SE has been linked to different hospital features, such as higher nurses’ independence and self-determination, control over resources, and good relationships with doctors [42,43]. While doing a job in an empowering work-atmosphere, the nursing staff has shared professional support and the proper resource allocation needed for good quality patient care and reduced unwanted patient outcomes [42,44]. SE has been considered an essential predictor of job satisfaction in the healthcare delivery system especially with nursing staff [45,46], nurses’ job engagement [47], a higher level of confidence, commitment and trust [30], intentions to leave [41], higher quality healthcare, patient’s preferred outcomes, and a decrease in APOs [48]. Scholars recommend that nurse-followers practicing under the supervision of transformational leaders can enjoy greater empowerment, which leads to higher JS and improved QOC outcomes, as a result [33,49]. Due to these factors, we made the following predictions.

**Hypothesis 3** **(H3).** *SE is positively related to nurse-assessed QOC.*

**Hypothesis 4** **(H4).** *Nurses’ JS is positively related to nurse-assessed QOC.*

**Hypothesis 5** **(H5).** *SE is negatively related to APO.*

**Hypothesis 6** **(H6).** *Nurses’ JS is negatively related to APO.*

### 2.3. The Mediating Role of Job Satisfaction and Structural Empowerment

JS is an essential nurse’s outcome that is highly influenced by the quality of the working atmosphere. Even though numerous studies have shown a greater JS among healthcare personnel, the existence of a higher level of job dissatisfaction among nurses is also significant [38,50]. The existing literature demonstrated a strong association between nurses’ JS and the quality of the working environment [45,51]. Abilities to meet with patients’ needs, professional opportunities, relationships with coworkers, and a balanced workload are the main work environment factors that influence nurses’ JS in the workplace [28,37]. The nurse’s JS plays a crucial role to encounter the challenges associated with patient’s satisfaction, nurses’ commitment to their institution, and a better quality of patients’ outcomes [7,46,50].

Previous studies revealed that an effective leadership style can help healthcare organizations to create a healthy environment in order to achieve the desired nurse-patient outcomes. In this regard, only limited quantitative research has been conducted that recognizes the direct and indirect processes that help the leaders to foster changes among their followers and enhance the level of patient satisfaction [15,27]. Therefore, we developed a theoretical model that illustrates the effects of TL on nurse-assessed QOC, and APO can be examined through SE and JS.

The perceptions of nurses regarding patients’ QOC improved with the time they spent with their patients and with their experience and expertise. These nurse-assessed QOC perceptions influence their job and career status, satisfaction and retention, and develop professional and practical behaviors [37,41,52]. Logically, these behaviors enhance nurse-assessed QOC and further enable the nursing staff to express these behaviors in a way that establishes a healthy practicing environment, which ultimately leads to positive patient outcomes and decreases the possibility of APOs [9,53].

Empowered and satisfied nurses that are highly committed to their jobs [54] also put in extra efforts towards their work [55] and possessed higher levels of QOC [39,49] Therefore, structurally empowered nurses are more obedient and loyal with the leaders, top-management, and the organizational policies and procedures implemented by the administration. These behaviors positively influence patient outcomes and QOC. 

Moreover, SE is also highly associated with the working conditions and the environment, which allow the nurses to practice professionally. Scholars have found that transformational leaders with the help of SE revealed that SE enhanced nurses’ autonomy level enables them to be involved in the decision making practices and provides them with easy access to the required information, resource allocation, and professional support [42,44,56,57]. According to Manojlovich [58], the positive collaboration between nurses and doctors can establish a better working environment. These collaborative practices play an intervening role between a structurally empowered work environment and nurse-assessed QOC and patient outcomes [49,59]. Based on the above evidence, we propose the following hypotheses.

**Hypothesis 7** **(H7).** *SE mediates the negative relationship between TL and APO.*

**Hypothesis 8** **(H8).** *JS mediates the negative relationship between TL and APO.*

**Hypothesis 9** **(H9).** *SE mediates the positive relationship between TL and QOC.*

**Hypothesis 10** **(H10).** *JS mediates the positive relationship between TL and QOC.*

## 3. Materials and Methods 

### 3.1. Sample and Design

A cross-sectional data with a random sampling technique was used to evaluate the hypotheses. The study was conducted in 17 government hospitals, which included 4 district headquarter hospitals and 13 tehsil headquarter hospitals situated in the Sargodha division, Punjab province, Pakistan, from March to April 2019. The data was collected from 600 registered female nurses who have a minimum of 1-year experience and are directly working under head nurses or nurse managers. After the participants’ desirability, the survey questionnaire was distributed to them and their secrecy was also assured. The questionnaire was divided into two parts. First part referred to the participants’ demographic profiles that included age, gender, education, experience, and employment status (see Table 1 for more details). In the second part, participants were asked to rate other factors, which included independent, dependent and mediating variables. 

A total number of 386 participants completed the survey with a response rate of 64.33%. Moreover, we applied two different tests, which included Harman’s one-factor test and the common latent factor (CLF), to overcome the possibility of method bias. The total variance explained during these tests is 22.57%, which is less than 50%, so the data is unbiased [60].

### 3.2. Measures

TL was evaluated using the 7 item scale developed by Carless, et al. [61]. A sample item for TL included my supervisor communicates a clear and positive vision of the future. The value of α for the TL scale was 0.97. This study adopted the 12 items’ *SE* scale proposed by Laschinger et al. [30]. A sample item for SE included the work is challenging for me. The reliability for this scale was 0.98. JS was evaluated using the 3 item scale proposed by Cammann [62]. A sample item for JS was all in all, I am satisfied with my job. The reliability (α) for this scale was 0.97. We adopted a 5 item scale to measure APO that was developed by Sochalski [53]. A sample item for APO was medication errors occur frequently. The reliability (α) for this scale was 0.93. A 4-item *QOC* scale was adopted in this study, which was developed by Aiken, et al. [63].A sample item was how do you evaluate the quality of the care you offer to patients. The reliability (α) for QOC scale was 0.94. All 31 items used in this study were measured on a 5-point Likert’s scale.

## 4. Results

### 4.1. Data Analysis

In order to analyze the data and the results, we used SPSS and AMOS version 25.0 (IBM, New York, NY, USA) as statistical tools. For testing mediation effects, structural equation modeling (SEM) with maximum likelihood estimation was employed [64,65]. We used different fit indices including chi-square (χ^2^), a comparative fit index (CFI), incremental fit index (IFI), Tucker-Lewis index (TLI), standardized root mean square residual (SRMR), and root meanssquare error of approximation (RMSEA) [64,66] to validate the model fit. According to Bentlerand Bonett [67], the values of χ^2^/df should be less than 3 for a model fit, and the values of CFI, IFI, and TLI should be greater than 0.90. [64]. The values of RMSEA and SRMR should not exceed 0.08 [68].

### 4.2. Demographic Characteristics

As previously discussed, this study contains only female participants (100%) and no male participants were part of the study. In terms of age, 69.17% of the participants (267 nurses) ranged from 21–30 years of age, and 0.78% of the participants were 51–60 years of age, which included only 3 nurses. Approximately 63.21% of the study participants (244 nurses) have a bachelors degree in nursing degree (BScN), and only 8 participants have masters degree (MScN) with a percentage of 2.07. Among the 386 participants, 77.20% were full-time employees and 22.80% were part-time. A total number of 241 (62.44%) participants have experience of 1-5 years, and only 3 participants (0.78%) have 31–35 years of experience (see Table 1 for more details).

### 4.3. Descriptive Statistics 

The values of the mean, SD, and correlations of all the studied variables are illustrated in Table 2. The mean values ranged from 2.91–3.38, and the values of SD ranged from 0.53–0.98. It can be seen in Table 2 that the correlations among TL, SE, JS, and QOC are positive and significant, but the correlation of TL, SE, JS, and QOC with APO are significant and negative. Table 2 further demonstrates the discriminant validity among all the constructs where the values of average variance extracted (AVE) are greater than the inter-correlational values [69].

### 4.4. Measurement Model

The measurement model is illustrated in Table 3, and the factor loadings, Cronbach’s α, the t-values values of AVE, and the composite reliabilities of all constructs are demonstrated. The alpha (α) coefficients for TL, SE, JS, APO, and QOC are 0.87, 0.82, 0.81, 0.78, and 0.81 respectively. These αs are above the recommended value of 0.70 [70]. The standardized factor loadings ranged from 0.72–0.88 for TL, from 0.74–0.85 for SE, from 0.79 0.84 for JS, from 0.73–0.85 for APO, and from 0.74–0.88 for QOC. All factor loadings are greater than 0.50 [70] and contribute significantly. The t-values for each item of all the studied variables are greater than 1.96 [64,71]. The values of average variance explained (AVE) for TL, SE, JS, APO, and QOC are 0.67, 0.64, 0.65, 0.66, and 0.63 respectively. These values provide convergent validity, because all of them are above the recommended value of 0.50 [72]. The composite reliability (CR) for TL, SE, JS, APO, and QOC ranged from 0.82–0.89 and are greater than the recommended criteria 0.60 [73].

### 4.5. Confirmatory Factor Analysis (CFA)

A CFA is the most important statistical test to ascertain the discriminant validity, especially for the mediation model. Therefore, we evaluated the discriminant validity of all the studied variables, which included TL, SE, JS, APO, and QOC, using AMOS 25.0 before the hypotheses testing. It can be seen in Table 4 that the 5-factor model has the best-fitted indices, because the values of χ^2^ = 479.13, χ^2^/df = 1.67, CFI = 0.98, IFI = 0.98, TLI = 0.97, SRMR = 0.03, and RMSEA = 0.034. We further established three alternative models that included two 4-factor models, which SE and JS are combined in the first model, and APO and QOC are combined in the second model, and a 1-factor model, which has all the items loaded on a single factor. After comparing all the models, we found the 5-factor model was the best-fitted for this study.

### 4.6. Hypotheses Testing

For testing hypotheses, we performed SEM with a maximum-likelihood estimate. The correlations among all constructs are illustrated in Table 2, and the regression coefficients (β) are illustrated in Table 5. H1 for our study is TL is positively related to nurses’ JS. We found support (r = 0.43, β = 0.37, *t* = 7.71, and *p* < 0.01) for Hypotheses 1 from Table 2 and Table 5. H2 is TL is positively related to SE. Table 2 and Table 5 provide the evidence for this hypothesis (r = 0.38, β = 0.41, *t* = 8.72, and *p* < 0.01). H3 predicted that SE is positively related to nurse-assessed QOC, and we found supportive evidence (r = 0.41, β = 0.43, *t* = 8.96, and *p* < 0.01) from Table 2 and Table 5. H4 was nurses’ JS is positively related to nurse-assessed QOC, and our results provided support (r = 0.36, β = 0.31, *t* = 6.08, and *p* < 0.01) as evident from Table 2 and Table 5. H5 was SE is negatively related to APO, and we found a negative association between SE and APO (r = −0.34, β = −0.32, *t* = −6.53, and *p* < 0.01). H6 predicted Nurses’ JS is negatively related to APO. A negative relation can be found in Table 2 and Table 5 where r = −0.24, β = −0.29, *t* = −6.04, and *p* < 0.01.

**Figure 1:**
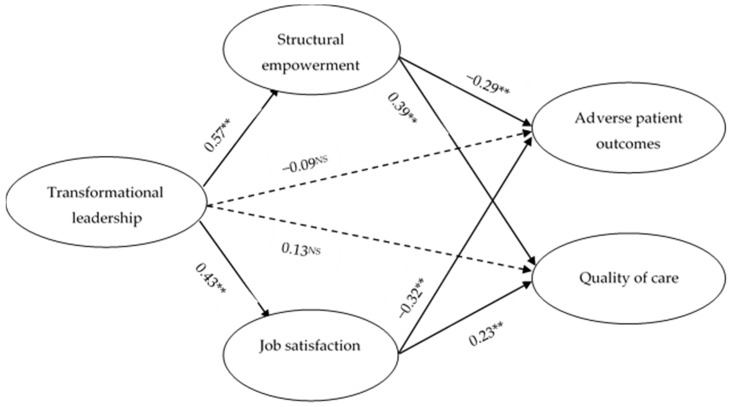
The results of the structural equation modeling (SEM) for mediation effects.

The results of the mediation effects using SEM are shown in Figure 1. The path from TL to SE is positive and significant (β = 0.57; *p* < 0.01), whereas the path from SE to APO is significantly negative (β = −0.29 and *p* < 0.01), and the path from SE to QOC is also positive and significant (β = 0.39 and *p* < 0.01). On the other hand, the path from TL to JS is significantly positive (β = 0.43 and *p* < 0.01), whereas the path from JS to APO is significant but negative in nature (β = −0.32 and *p* < 0.01), and the path to QOC is significantly positive (β = 0.23 and *p* < 0.01). It can be seen in Figure 1 that both direct paths from TL to APO (β = −0.09 and *p* > 0.05) and TL to QOC (β = 0.13 and *p* > 0.05) are insignificant and confirm full mediation. This evidence proves mediation Hypotheses 7–10.

## 5. Discussion and Implication

Decent work, availability of trained staff, and healthy work conditions in the health sector are the fundamental subject to ensuring effective and resilient health systems worldwide. The health sector is essentially about people; without health workers there can be no health care. To address health workforce shortages effectively, investments in the health workforce have to extend beyond increasing numbers of workers. More employment opportunities in the health sector are needed and those have to be accompanied by measures to provide decent employment and conditions of work in order to attract and retain competent health workers. The global context of the health sector has in recent years been marked by important initiatives for strengthening health systems and the health workforce. A common feature of these initiatives is the call for transformative action, and changing mindsets to seize the opportunities at hand and address the immense challenges ahead [74].

In this context, the main aim of this research was to explore the influence of TL on nurse-assessed APO and QOC through the mediating effects of SE and JS. The key finding of our study was to test the significant mediating mechanism of SE and JS on the relationship between TL and both APO and QOC. Though TL plays a vital role in providing nurses an empowered working environment to improve patient outcomes [14], limited research has been conducted to test the influence of TL on SE. Prior research revealed the association between TL and empowerment with the perspective of a psychological conceptualization [75,76].

The results of our study indicate that nursing leaders who exhibit transformational behavior significantly predict the desired patient’s outcomes by reducing the possibility of adverse patient outcomes (APOs) and increase quality of care (QOC) through the intervening influence of structural empowerment (SE) and job satisfaction (JS). To be consistent with previous research, our study provides positive results since adopting the effective leadership style and intervening variables for improving patient outcomes face some complications. For example, Higgins [28] discovered a negative association between nurse transformational managers and APOs through the intervention of organizational citizenship behavior (OCB). In another study, the authors exposed a significant association between authentic leadership (AL) and reduced nurse-assessed APOs using trust in the leader as a mediator [77]. In our study, the perception of the nurses regarding their leaders was highly transformational.

Transformational leaders express a higher level of expectations towards their subordinates and motivate them to exert extra efforts to achieve the organizational vision. This will also increase their level of commitment [24,78]. Transformational leaders also have the abilities of charismatic leadership, which enables them to inspire their followers to work beyond the expectations. To become successful, these leaders clearly communicate the organizational mission to the employees, provide them a clear direction and support, link their work with organizational goals, and influence their level of commitment in a positive way.

In the healthcare sector, transformational leaders seek out the needs of the nurses and establish a strong relationship with them as a mentor or coach, listen to their problems, and promote a healthy work environment for continuous development [21]. When transformational leaders take an interest in nursing development and empower them to utilize their full potential, nurses feel more self-confidence and work engagement, which ultimately leads to enhanced patients’ QOC.

Our findings are consistent with previous research that TL may affect the occurrence of adverse events, since transformational leaders believe in evidence-based work and influence employees to offer innovative ideas to solve the problems [21,79]. This may increase the nurses’ level of satisfaction and empowerment and make nurses responsible to take better care of patients with fewer medical errors. In addition, the results of the present study revealed that accessibility to information, the availability of resources, and the decision-making participation make the nurses more satisfied and trustworthy. The strong relationships between TL, SE, and JS signify that improved healthy work conditions and environment could be the most important element to improve QOC and decrease APO and job effectiveness. By establishing deep relationships, transformational leaders recognize and foresee the basic necessities of their nurses and make significant efforts to fulfill their needs and allocate proper resources to encourage a sense of empowerment and autonomy, which results in improved desired patient outcomes and satisfaction.

## 6. Limitations

This study also exhibits some limitations. First, this research is based on a cross-sectional nature, which restricts the interpretation of the evidence-based causal relationships and theoretical foundations among the studied variables [80]. A longitudinal study should be conducted in the future to examine the leaders’ TL and its impact on patient satisfaction, nurses’ outcomes, and the work environment. Second, our study focused on only two mediators (SE and JS), and future research should opt to use other intervening variables, such as safety climate, commitment, and burnout, to comprehensively understand the influence of TL on healthcare outcomes. Third, we recruited only female nurse participants [81,82]. Future research should include male nurses as well. Fourth, our study used self-reported instruments, which may cause a response bias [83]. We used Herman’s one-factor and CLF techniques to test the possibility of this biasness, in future studies; it is recommended to keep it in mind. Finally, our study sample consisted of female nurses who were working in government hospitals, and future studies should incorporate both public and private hospitals with a wider area. 

## 7. Conclusions

The results of the present study demonstrated the significant role that transformational leadership played to enhance the working conditions and atmosphere, which allows the nurses to establish good relations with patients and improve quality of care and patient satisfaction. Our findings, empirically contribute to the existing literature by establishing a strong and evidence-based relationship between transformational leadership and nurse-assessed quality of care and adverse patient outcomes. Second, we found structural empowerment and job satisfaction as the potential mediators for these relationships. Our findings further suggest that transformational leadership is a vital indicator that can help the healthcare organizations to improve quality of care, nurses’ job satisfaction, and reduce adverse patient outcomes. To overcome the occurrence of adverse events in healthcare organizations, it is recommended that top management should develop transformational leadership behaviors among nurse managers/leaders and provide such environments empowerment and autonomy in order to support their follower nurses. This will better lead to the desired outcomes and improve quality of care.

## Figures and Tables

**Table 1:** Demographic Characteristics.

Demographic Characteristics		Number (*n*)	%
Gender			
	Female	386	100
	Male	0	0
Age (years)			
	21–30	267	69.17
	31–40	81	20.98
	41–50	35	9.07
	51–60	3	0.78
Education			
	Nursing Diploma	53	13.73
	Associate Degree	81	20.98
	Bachelor	244	63.21
	Master	8	2.07
Employment Status			
	Full time	298	77.20
	Part time	88	22.80
Experience (years)			
	1–5	241	62.44
	6–10	87	22.54
	11–15	23	5.96
	16–20	14	3.63
	21–25	10	2.59
	26–30	8	2.07
	31–35	3	0.78

Note 1: bachelor degree is enough for nurses to work in Pakistan.

**Table 2:** Descriptive statistics and correlations.

*n* = 386	Mean	SD	Correlations				
			1	2	3	4	5
1. TL	3.38	0.98	**(0.82)**				
2. SE	2.97	0.53	0.38 **	**(0.80)**			
3. JS	3.13	0.81	0.43 **	0.48 **	**(0.81)**		
4. QOC	2.80	0.76	0.30 **	0.41 **	0.36 **	**(0.79)**	
5. APO	2.91	0.63	−0.26 **	−0.34 **	−0.29 **	−0.24 **	**(0.81)**

Significance (2-tailed): ** *p* < 0.01. Square root of AVE is shown in parenthesis (bold) demonstrating discriminant validity. Note: SD: standard deviation; TL: transformational leadership; SE: structural empowerment; JS: job satisfaction; QOC: quality of care; APO: adverse patient outcomes; AVE: average variance extracted.

**Table 3:** Overall measurement model.

Construct	Cronbach’s α	Items	Stand. Factor Loadings	S.E.	T	AVE	Composite Reliability (CR)
TL	0.87	TL1	0.84	-	-	0.67	0.89
		TL2	0.85	0.051	16.67		
		TL3	0.78	0.053	14.72		
		TL4	0.72	0.050	14.40		
		TL5	0.83	0.053	15.66		
		TL6	0.88	0.054	16.30		
		TL7	0.87	0.054	16.11		
SE	0.82	SE1	0.83	-		0.64	0.85
		SE2	0.85	0.052	16.35		
		SE3	0.79	0.051	15.49		
		SE4	0.81	0.052	15.58		
		SE5	0.76	0.051	14.90		
		SE6	0.81	0.051	15.88		
		SE7	0.83	0.052	15.96		
		SE8	0.85	0.051	16.67		
		SE9	0.79	0.050	15.80		
		SE10	0.78	0.050	15.60		
		SE11	0.74	0.052	14.23		
		SE12	0.82	0.051	16.08		
JS	0.81	JS1	0.84	-		0.65	0.84
		JS2	0.79	0.052	15.19		
		JS3	0.80	0.051	15.69		
APO	0.78	APO1	0.81	-		0.66	0.82
		APO2	0.73	0.050	14.60		
		APO3	0.78	0.051	15.29		
		APO4	0.85	0.052	16.35		
		APO5	0.82	0.051	16.08		
QOC	0.81	QOC1	0.88	-		0.63	0.83
		QOC2	0.83	0.053	15.66		
		QOC3	0.76	0.051	14.90		
		QOC4	0.74	0.051	14.51		

**Table 4:** Confirmatory Factor Analysis (CFA).

Model	χ^2^	df	χ^2^/df	CFI	IFI	TLI	SRMR	RMSEA
5-Factor model	479.13	287	1.67	0.98	0.98	0.97	0.03	0.034
4-Factor model (SE + JS combined)	653.39	302	2.16	0.93	0.93	0.94	0.08	0.059
4-Factor model (APO + QOC combined)	598.27	302	1.98	0.96	0.95	0.90	0.06	0.052
1-Factor model	4557.69	293	15.56	0.49	0.50	0.48	0.27	0.195

Note: SE: structural empowerment; JS: job satisfaction; APO: adverse patient outcomes; QOC: quality of care; df: degree of freedom; CFI: comparative fit index; IFI: incremental fit index; TLI: Tucker-Lewis index; SRMR: standardized root-mean-square residual; RMSEA: root-mean-square error of approximation.

**Table 5:** β coefficients for testing hypotheses 1–6.

Path	Standardized β	S.E.	T	*p*-value	Significance
TL 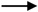 JS	0.37	0.048	7.71	<0.01	(**)
TL 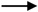 SE	0.41	0.047	8.72	<0.01	(**)
SE 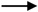 QOC	0.43	0.048	8.96	<0.01	(**)
JS 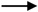 QOC	0.31	0.051	6.08	<0.01	(**)
SE 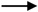 APO	−0.32	0.049	−6.53	<0.01	(**)
JS 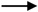 APO	−0.29	0.048	−6.04	<0.01	(**)

Note: TL: transformational leadership; JS: job satisfaction; SE: structural empowerment; QOC: quality of care; APO: adverse patient outcomes; S.E.: standard error.

## Abbreviations

TL	transformational leadership
SE	structural empowerment
JS	job satisfaction
APO	adverse patient outcomes
QOC	quality of care
